# Diaqua­bis­[4-(4*H*-1,2,4-triazol-4-yl)benzoato-κ^2^ *O*,*O*′]nickel(II)

**DOI:** 10.1107/S1600536811016643

**Published:** 2011-05-07

**Authors:** Shuzhi Xu, Wenxin Shao, Miao Yu, Guihua Gong

**Affiliations:** a Jilin Business and Technology College, Changchun 130062, People’s Republic of China

## Abstract

In the title compound, [Ni(C_9_H_6_N_3_O_2_)_2_(H_2_O)_2_], the Ni^II^ atom lies on a twofold rotation axis and is six-coordinated by two bidentate chelating 4-(1,2,4-triazol-4-yl)benzoate ligands and two water mol­ecules in a distorted octa­hedral geometry. Inter­molecular O—H⋯N hydrogen bonds link the complex mol­ecules into a two-dimensional network parallel to (010).

## Related literature

For general background to the structures and applications of metal complexes, see: Mahata *et al.* (2009 ▶); Perry *et al.* (2004 ▶); Qin *et al.* (2005 ▶); Shi *et al.* (2009 ▶). For a related structure, see: Zhu (2010 ▶).
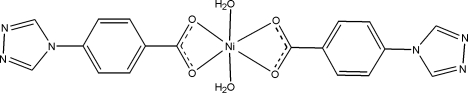


## Experimental

### 

#### Crystal data

[Ni(C_9_H_6_N_3_O_2_)_2_(H_2_O)_2_]
*M* *_r_* = 471.06
Monoclinic, 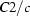

*a* = 13.5194 (6) Å
*b* = 9.8480 (5) Å
*c* = 14.3234 (7) Å
β = 112.293 (1)°
*V* = 1764.47 (15) Å^3^
*Z* = 4
Mo *K*α radiation
μ = 1.16 mm^−1^
*T* = 296 K
0.28 × 0.24 × 0.22 mm

#### Data collection

Bruker APEXII CCD diffractometer
Absorption correction: multi-scan (*SADABS*; Bruker, 2001 ▶) *T*_min_ = 0.72, *T*_max_ = 0.82
4732 measured reflections
1744 independent reflections
1662 reflections with *I* > 2σ(*I*)
*R*_int_ = 0.021

#### Refinement

*R*[*F* ^2^ > 2σ(*F* ^2^)] = 0.030
*wR*(*F* ^2^) = 0.084
*S* = 1.13
1744 reflections
147 parameters
2 restraints
H atoms treated by a mixture of independent and constrained refinement
Δρ_max_ = 0.31 e Å^−3^
Δρ_min_ = −0.45 e Å^−3^

### 

Data collection: *APEX2* (Bruker, 2007 ▶); cell refinement: *SAINT* (Bruker, 2007 ▶); data reduction: *SAINT*; program(s) used to solve structure: *SHELXTL* (Sheldrick, 2008 ▶); program(s) used to refine structure: *SHELXTL*; molecular graphics: *SHELXTL* and *Mercury* (Macrae *et al.*, 2006 ▶); software used to prepare material for publication: *SHELXTL*.

## Supplementary Material

Crystal structure: contains datablocks global, I. DOI: 10.1107/S1600536811016643/hy2427sup1.cif

Structure factors: contains datablocks I. DOI: 10.1107/S1600536811016643/hy2427Isup2.hkl

Additional supplementary materials:  crystallographic information; 3D view; checkCIF report

## Figures and Tables

**Table 1:** Selected bond lengths (Å)

Ni1—O1	2.1507 (14)
Ni1—O2	2.1240 (14)
Ni1—O3	2.0453 (16)

**Table 2:** Hydrogen-bond geometry (Å, °)

*D*—H⋯*A*	*D*—H	H⋯*A*	*D*⋯*A*	*D*—H⋯*A*
O3—H3*B*⋯N2^i^	0.78 (2)	2.06 (2)	2.836 (2)	172 (3)
O3—H3*A*⋯N3^ii^	0.79 (2)	1.99 (2)	2.768 (2)	169 (3)

Symmetry codes: (i) 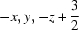; (ii) 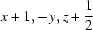.

**Fig. 1.**
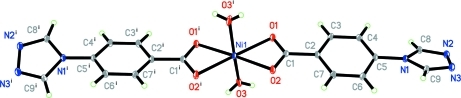
Molecular structure of the title compound. Displacement ellipsoids are drawn at the 30% probability level. [Symmetry code: (i) 1-x, y, 3/2-z.]

**Fig. 2.**
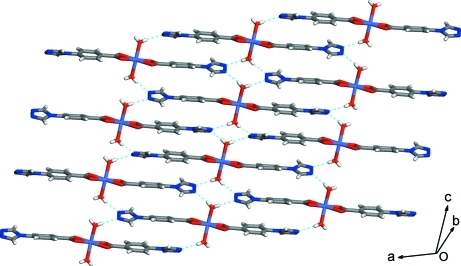
View of the layer structure in the title compound, built by O—H···N hydrogen bonds (dashed lines).

## supplementary crystallographic information

### Comment

The construction of novel coordination complexes is the current interest in the field of supramolecular chemistry and crystal engineering stemming from their potential applications as functional materials, as well as their intriguing variety of architectures and topologies (Perry *et al.*, 2004; Qin *et al.*, 2005). Heterocyclic carboxylates have often been used as mono-, bi- or multi-dentate ligands to bind transition metal centers, leading to the formation of moderately robust metal–organic coordination frameworks (Mahata *et al.*, 2009; Shi *et al.*, 2009). In this contribution, we selected 4-(1,2,4-triazol-4-yl)benzoic acid (Htyb) as an organic carboxylate ligand, generating the title compound, which is reported here.

In the title compound, the Ni^II^ atom lies on a twofold rotation axis and adopts a distorted octahedral coordination geometry, being coordinated by four carboxylate O atoms from two tyb ligands and two water molecules (Fig. 1, Table 1). The Ni—O bond lengths and the O—Ni—O bond angles are in the normal range (Zhu, 2010). Intermolecular O—H···N hydrogen bonds (Table 2) stabilize the structure and give a two-dimensional network (Fig. 2).

### Experimental

The synthesis was performed under hydrothermal conditions. A mixture of Ni(CH_3_COO)_2_.4H_2_O (0.2 mmol, 0.05 g), 4-(1,2,4-triazol-4-yl)benzoic acid (0.4 mmol, 0.075 g), NaOH (0.4 mmol, 0.016 g) and H_2_O (15 ml) in a 25 ml stainless steel reactor with a Teflon liner was heated from 293 to 443 K in 2 h and a constant temperature was maintained at 443 K for 72 h. After the mixture was cooled to 298 K, green crystals of the title compound were obtained.

### Refinement

H atoms on C atoms were positioned geometrically and refined as riding atoms, with C—H = 0.93 Å and with *U*_iso_(H) = 1.2*U*_eq_(C). H atoms bonded to water O atom were located in a difference Fourier map and refined with a restraint of O—H = 0.85 (1) Å.

### Crystal data

**Table:**

[Ni(C_9_H_6_N_3_O_2_)_2_(H_2_O)_2_]	*F*(000) = 968
*M**_r_* = 471.06	*D*_x_ = 1.773 Mg m^−^^3^
Monoclinic, *C*2/*c*	Mo *K*α radiation, λ = 0.71073 Å
Hall symbol: -C 2yc	Cell parameters from 1744 reflections
*a* = 13.5194 (6) Å	θ = 1.0–26.0°
*b* = 9.8480 (5) Å	µ = 1.16 mm^−^^1^
*c* = 14.3234 (7) Å	*T* = 296 K
β = 112.293 (1)°	Block, green
*V* = 1764.47 (15) Å^3^	0.28 × 0.24 × 0.22 mm
*Z* = 4	

### Data collection

**Table:**

Bruker APEXII CCD diffractometer	1744 independent reflections
Radiation source: fine-focus sealed tube	1662 reflections with *I* > 2σ(*I*)
graphite	*R*_int_ = 0.021
φ and ω scans	θ_max_ = 26.0°, θ_min_ = 2.6°
Absorption correction: multi-scan (*SADABS*; Bruker, 2001)	*h* = −16→16
*T*_min_ = 0.72, *T*_max_ = 0.82	*k* = −12→9
4732 measured reflections	*l* = −14→17

### Refinement

**Table:**

Refinement on *F*^2^	Primary atom site location: structure-invariant direct methods
Least-squares matrix: full	Secondary atom site location: difference Fourier map
*R*[*F*^2^ > 2σ(*F*^2^)] = 0.030	Hydrogen site location: inferred from neighbouring sites
*wR*(*F*^2^) = 0.084	H atoms treated by a mixture of independent and constrained refinement
*S* = 1.13	*w* = 1/[σ^2^(*F*_o_^2^) + (0.0345*P*)^2^ + 4.2534*P*] where *P* = (*F*_o_^2^ + 2*F*_c_^2^)/3
1744 reflections	(Δ/σ)_max_ < 0.001
147 parameters	Δρ_max_ = 0.31 e Å^−^^3^
2 restraints	Δρ_min_ = −0.45 e Å^−^^3^

### Fractional atomic coordinates and isotropic or equivalent isotropic displacement parameters (Å^2^)

**Table:**

	*x*	*y*	*z*	*U*_iso_*/*U*_eq_	
C1	0.30391 (16)	0.07262 (19)	0.68990 (15)	0.0139 (4)	
C2	0.18541 (15)	0.0695 (2)	0.66363 (15)	0.0144 (4)	
C3	0.12703 (16)	0.1900 (2)	0.64420 (15)	0.0164 (4)	
H3	0.1616	0.2723	0.6465	0.020*	
C4	0.01757 (15)	0.1882 (2)	0.62145 (15)	0.0164 (4)	
H4	−0.0217	0.2684	0.6074	0.020*	
C5	−0.03212 (15)	0.0645 (2)	0.62014 (15)	0.0132 (4)	
C6	0.02504 (16)	−0.0562 (2)	0.64143 (16)	0.0167 (4)	
H6	−0.0092	−0.1381	0.6416	0.020*	
C7	0.13424 (16)	−0.0531 (2)	0.66248 (16)	0.0163 (4)	
H7	0.1732	−0.1336	0.6759	0.020*	
C8	−0.21097 (15)	0.1595 (2)	0.60494 (15)	0.0157 (4)	
H8	−0.1890	0.2466	0.6290	0.019*	
C9	−0.21064 (16)	−0.0489 (2)	0.55868 (16)	0.0178 (4)	
H9	−0.1883	−0.1332	0.5450	0.021*	
N1	−0.14499 (13)	0.05953 (17)	0.59661 (12)	0.0136 (4)	
N2	−0.30914 (13)	0.11583 (19)	0.57442 (13)	0.0176 (4)	
N3	−0.30856 (13)	−0.01823 (19)	0.54433 (13)	0.0188 (4)	
O1	0.35080 (11)	0.18546 (14)	0.69937 (11)	0.0162 (3)	
O2	0.35618 (11)	−0.03741 (15)	0.70360 (11)	0.0184 (3)	
O3	0.50303 (12)	0.10080 (19)	0.89291 (12)	0.0248 (4)	
Ni1	0.5000	0.07701 (4)	0.7500	0.01674 (14)	
H3A	0.5529 (17)	0.080 (3)	0.9416 (16)	0.025*	
H3B	0.4511 (17)	0.113 (3)	0.903 (2)	0.025*	

### Atomic displacement parameters (Å^2^)

**Table:**

	*U*^11^	*U*^22^	*U*^33^	*U*^12^	*U*^13^	*U*^23^
C1	0.0112 (10)	0.0169 (10)	0.0139 (9)	−0.0001 (7)	0.0050 (8)	−0.0002 (7)
C2	0.0097 (10)	0.0186 (10)	0.0148 (9)	0.0000 (7)	0.0045 (8)	−0.0001 (7)
C3	0.0125 (9)	0.0150 (10)	0.0218 (10)	−0.0022 (8)	0.0066 (8)	0.0014 (8)
C4	0.0114 (9)	0.0149 (10)	0.0221 (10)	0.0024 (7)	0.0056 (8)	0.0033 (8)
C5	0.0073 (9)	0.0189 (10)	0.0132 (9)	−0.0003 (7)	0.0038 (7)	−0.0006 (7)
C6	0.0122 (10)	0.0153 (10)	0.0229 (11)	−0.0022 (8)	0.0069 (8)	−0.0005 (8)
C7	0.0124 (10)	0.0142 (9)	0.0229 (10)	0.0018 (8)	0.0075 (8)	0.0003 (8)
C8	0.0104 (9)	0.0193 (10)	0.0174 (10)	0.0019 (8)	0.0053 (8)	0.0006 (8)
C9	0.0135 (10)	0.0188 (10)	0.0204 (10)	−0.0034 (8)	0.0056 (8)	−0.0026 (8)
N1	0.0087 (8)	0.0164 (8)	0.0156 (8)	−0.0013 (6)	0.0045 (7)	−0.0001 (6)
N2	0.0110 (8)	0.0230 (9)	0.0183 (9)	−0.0001 (7)	0.0049 (7)	0.0005 (7)
N3	0.0118 (8)	0.0232 (9)	0.0203 (9)	−0.0025 (7)	0.0047 (7)	−0.0003 (7)
O1	0.0089 (6)	0.0161 (7)	0.0231 (7)	−0.0010 (5)	0.0054 (6)	0.0001 (6)
O2	0.0091 (6)	0.0164 (7)	0.0293 (8)	0.0008 (6)	0.0070 (6)	0.0005 (6)
O3	0.0090 (7)	0.0468 (10)	0.0188 (8)	0.0075 (7)	0.0054 (6)	0.0057 (7)
Ni1	0.0100 (2)	0.0175 (2)	0.0222 (2)	0.000	0.00558 (16)	0.000

### Geometric parameters (Å, °)

**Table:**

C1—O1	1.261 (2)	C7—H7	0.9300
C1—O2	1.268 (2)	C8—N2	1.303 (3)
C1—C2	1.501 (3)	C8—N1	1.364 (3)
C1—Ni1	2.458 (2)	C8—H8	0.9300
C2—C7	1.388 (3)	C9—N3	1.296 (3)
C2—C3	1.394 (3)	C9—N1	1.363 (3)
C3—C4	1.389 (3)	C9—H9	0.9300
C3—H3	0.9300	N2—N3	1.390 (3)
C4—C5	1.388 (3)	O3—H3A	0.79 (2)
C4—H4	0.9300	O3—H3B	0.78 (2)
C5—C6	1.387 (3)	Ni1—O1	2.1507 (14)
C5—N1	1.433 (2)	Ni1—O2	2.1240 (14)
C6—C7	1.391 (3)	Ni1—O3	2.0453 (16)
C6—H6	0.9300		
			
O1—C1—O2	120.58 (18)	C9—N3—N2	107.32 (17)
O1—C1—C2	119.38 (17)	C1—O1—Ni1	88.14 (11)
O2—C1—C2	120.03 (17)	C1—O2—Ni1	89.16 (12)
O1—C1—Ni1	61.01 (10)	Ni1—O3—H3A	123 (2)
O2—C1—Ni1	59.79 (10)	Ni1—O3—H3B	122 (2)
C2—C1—Ni1	174.50 (14)	H3A—O3—H3B	114 (3)
C7—C2—C3	119.77 (18)	O3—Ni1—O3^i^	166.84 (11)
C7—C2—C1	120.05 (18)	O3—Ni1—O2	92.43 (6)
C3—C2—C1	120.14 (17)	O3^i^—Ni1—O2	94.54 (6)
C4—C3—C2	120.52 (18)	O3—Ni1—O2^i^	94.54 (6)
C4—C3—H3	119.7	O3^i^—Ni1—O2^i^	92.43 (6)
C2—C3—H3	119.7	O2—Ni1—O2^i^	115.92 (8)
C5—C4—C3	118.77 (18)	O3—Ni1—O1^i^	86.88 (6)
C5—C4—H4	120.6	O3^i^—Ni1—O1^i^	86.60 (6)
C3—C4—H4	120.6	O2—Ni1—O1^i^	177.55 (6)
C6—C5—C4	121.53 (18)	O2^i^—Ni1—O1^i^	61.82 (6)
C6—C5—N1	118.50 (17)	O3—Ni1—O1	86.60 (6)
C4—C5—N1	119.97 (17)	O3^i^—Ni1—O1	86.88 (6)
C5—C6—C7	119.07 (18)	O2—Ni1—O1	61.82 (6)
C5—C6—H6	120.5	O2^i^—Ni1—O1	177.55 (6)
C7—C6—H6	120.5	O1^i^—Ni1—O1	120.45 (8)
C2—C7—C6	120.32 (19)	O3—Ni1—C1	87.82 (6)
C2—C7—H7	119.8	O3^i^—Ni1—C1	92.41 (6)
C6—C7—H7	119.8	O2—Ni1—C1	31.05 (6)
N2—C8—N1	110.45 (18)	O2^i^—Ni1—C1	146.94 (6)
N2—C8—H8	124.8	O1^i^—Ni1—C1	151.16 (6)
N1—C8—H8	124.8	O1—Ni1—C1	30.85 (6)
N3—C9—N1	110.64 (19)	O3—Ni1—C1^i^	92.41 (6)
N3—C9—H9	124.7	O3^i^—Ni1—C1^i^	87.82 (6)
N1—C9—H9	124.7	O2—Ni1—C1^i^	146.94 (6)
C9—N1—C8	104.57 (17)	O2^i^—Ni1—C1^i^	31.05 (6)
C9—N1—C5	126.49 (17)	O1^i^—Ni1—C1^i^	30.85 (6)
C8—N1—C5	128.93 (17)	O1—Ni1—C1^i^	151.16 (6)
C8—N2—N3	107.02 (16)	C1—Ni1—C1^i^	177.99 (9)
			
O1—C1—C2—C7	−173.79 (19)	N1—C9—N3—N2	−0.5 (2)
O2—C1—C2—C7	5.3 (3)	C8—N2—N3—C9	0.4 (2)
O1—C1—C2—C3	3.7 (3)	O2—C1—O1—Ni1	−5.41 (19)
O2—C1—C2—C3	−177.18 (18)	C2—C1—O1—Ni1	173.72 (16)
C7—C2—C3—C4	−1.4 (3)	O1—C1—O2—Ni1	5.47 (19)
C1—C2—C3—C4	−178.94 (18)	C2—C1—O2—Ni1	−173.65 (16)
C2—C3—C4—C5	1.1 (3)	C1—O2—Ni1—O3	81.70 (12)
C3—C4—C5—C6	0.3 (3)	C1—O2—Ni1—O3^i^	−87.13 (12)
C3—C4—C5—N1	−179.81 (17)	C1—O2—Ni1—O2^i^	177.95 (13)
C4—C5—C6—C7	−1.4 (3)	C1—O1—Ni1—O3	−91.44 (12)
N1—C5—C6—C7	178.78 (18)	C1—O1—Ni1—O3^i^	100.00 (12)
C3—C2—C7—C6	0.4 (3)	C1—O1—Ni1—O2	3.15 (11)
C1—C2—C7—C6	177.89 (18)	C1—O1—Ni1—O1^i^	−175.80 (12)
C5—C6—C7—C2	1.0 (3)	O1—C1—Ni1—O3	87.02 (12)
N3—C9—N1—C8	0.4 (2)	O2—C1—Ni1—O3	−98.36 (12)
N3—C9—N1—C5	−178.76 (17)	O1—C1—Ni1—O3^i^	−79.81 (12)
N2—C8—N1—C9	−0.1 (2)	O2—C1—Ni1—O3^i^	94.80 (12)
N2—C8—N1—C5	178.99 (18)	O1—C1—Ni1—O2	−174.61 (19)
C6—C5—N1—C9	−24.4 (3)	O1—C1—Ni1—O2^i^	−178.00 (10)
C4—C5—N1—C9	155.7 (2)	O2—C1—Ni1—O2^i^	−3.4 (2)
C6—C5—N1—C8	156.7 (2)	O1—C1—Ni1—O1^i^	7.5 (2)
C4—C5—N1—C8	−23.2 (3)	O2—C1—Ni1—O1^i^	−177.86 (11)
N1—C8—N2—N3	−0.2 (2)	O2—C1—Ni1—O1	174.61 (19)

Symmetry codes: (i) −*x*+1, *y*, −*z*+3/2.

### Hydrogen-bond geometry (Å, °)

**Table:**

*D*—H···*A*	*D*—H	H···*A*	*D*···*A*	*D*—H···*A*
O3—H3B···N2^ii^	0.78 (2)	2.06 (2)	2.836 (2)	172 (3)
O3—H3A···N3^iii^	0.79 (2)	1.99 (2)	2.768 (2)	169 (3)

Symmetry codes: (ii) −*x*, *y*, −*z*+3/2; (iii) *x*+1, −*y*, *z*+1/2.
